# The circadian clock protein REVERBα inhibits pulmonary fibrosis development

**DOI:** 10.1073/pnas.1912109117

**Published:** 2019-12-26

**Authors:** Peter S. Cunningham, Peter Meijer, Alicja Nazgiewicz, Simon G. Anderson, Lee A. Borthwick, James Bagnall, Gareth B. Kitchen, Monika Lodyga, Nicola Begley, Rajamiyer V. Venkateswaran, Rajesh Shah, Paul F. Mercer, Hannah J. Durrington, Neil C. Henderson, Karen Piper-Hanley, Andrew J. Fisher, Rachel C. Chambers, David A. Bechtold, Julie E. Gibbs, Andrew S. Loudon, Martin K. Rutter, Boris Hinz, David W. Ray, John F. Blaikley

**Affiliations:** ^a^Faculty of Biology, Medicine and Health, The University of Manchester, Manchester M13 9PL, United Kingdom;; ^b^The George Alleyne Chronic Disease Research Centre, The University of the West Indies, Bridgetown. Barbados BB11000;; ^c^Fibrosis Research Group, Biosciences Institute, Newcastle University, Newcastle upon Tyne NE2 4HH, United Kingdom;; ^d^Manchester University National Health Service Foundation Trust, Manchester Academic Health Science Centre, Manchester M13 9WL, United Kingdom;; ^e^Laboratory of Tissue Repair and Regeneration, Faculty of Dentistry, University of Toronto, Toronto, ON M5G 1G6, Canada;; ^f^Centre for Inflammation and Tissue Repair, Faculty of Medical Sciences, University College London, London WC1E 6JJ, United Kingdom;; ^g^Centre for Inflammation Research, University of Edinburgh, EH16 4TJ Edinburgh, United Kingdom;; ^h^Institute of Transplantation, Freeman Hospital, The Newcastle upon Tyne Hospitals National Health Service Foundation Trust, Newcastle upon Tyne NE7 7DN, United Kingdom;; ^i^Translational and Clinical Research Institute, Newcastle University, Newcastle upon Tyne NE2 4HH, United Kingdom;; ^j^National Institute for Health Research Oxford Biomedical Research Centre, John Radcliffe Hospital, Oxford OX3 9DU, United Kingdom;; ^k^Oxford Centre for Diabetes, Endocrinology and Metabolism, University of Oxford, Oxford OX3 7LE, United Kingdom

**Keywords:** pulmonary fibrosis, circadian, Reverb alpha, sleep, integrin

## Abstract

Pulmonary inflammatory responses lie under circadian control; however, the importance of circadian mechanisms in the underlying fibrotic phenotype is not understood. Here, we identify a striking change to these mechanisms resulting in a gain of amplitude and lack of synchrony within pulmonary fibrotic tissue. These changes result from an infiltration of mesenchymal cells, an important cell type in the pathogenesis of pulmonary fibrosis. Mutation of the core clock protein REVERBα in these cells exacerbated the development of bleomycin-induced fibrosis, whereas mutation of REVERBα in club or myeloid cells had no effect on the bleomycin phenotype. Knockdown of REVERBα revealed regulation of the little-understood transcription factor TBPL1. Both REVERBα and TBPL1 altered integrinβ1 focal-adhesion formation, resulting in increased myofibroblast activation. The translational importance of our findings was established through analysis of 2 human cohorts. In the UK Biobank, circadian strain markers (sleep length, chronotype, and shift work) are associated with pulmonary fibrosis, making them risk factors. In a separate cohort, REVERBα expression was increased in human idiopathic pulmonary fibrosis (IPF) lung tissue. Pharmacological targeting of REVERBα inhibited myofibroblast activation in IPF fibroblasts and collagen secretion in organotypic cultures from IPF patients, thus suggesting that targeting of REVERBα could be a viable therapeutic approach.

## Significance

The circadian clock plays an essential role in energy metabolism and inflammation. In contrast, the importance of the clock in the pathogenesis of fibrosis remains poorly explored. This study describes a striking alteration in circadian biology during pulmonary fibrosis where the relatively arrhythmic alveolar structures gain circadian but asynchronous rhythmicity due to infiltration by fibroblasts. Disruption of the clock in these cells, which are not widely implicated in circadian pathophysiology, results in a profibrotic phenotype. Translation of these findings in humans revealed previously unrecognized important circadian risk factors for pulmonary fibrosis (sleep length, chronotype, and shift work). In addition, targeting REVERBα repressed collagen secretion from human fibrotic lung tissue, making this protein a promising therapeutic target.

The circadian clock in the lung drives important physiological responses, including temporal gating of a number of inflammatory (1–3) and antioxidant responses (4). Key cell types that are known to be important are the nonciliated, bronchial epithelial cells (club cells) (2) and alveolar macrophages (3, 5). In contrast, alveolar structures typically exhibit weak circadian oscillations (6). Genetic disruption of the *Clock* gene (4) impairs circadian pulmonary oscillations and leads to exaggerated pulmonary responses to bleomycin challenge, a model of pulmonary fibrosis (7).

Pulmonary fibrosis, including idiopathic pulmonary fibrosis (IPF), is frequently fatal with existing treatments slowing progression rather than curing the disease (8). The causes and nongenetic risk factors for IPF are poorly understood, with several studies implicating age, sex, smoking, and more recently air pollution (9). IPF is characterized histologically by the development of fibroblastic foci in the lung parenchyma (10). Cells in these foci are typically activated myofibroblasts (11) derived from multiple sources (12, 13), including pulmonary fibroblasts and pericytes (11, 14). Myofibroblasts secrete collagen, resulting in abnormal lung function and are characterized by increased focal-adhesion formation and acquisition of a contractile cytoskeleton with alpha smooth muscle actin (αSMA)-positive stress fibers (15). In addition to fibroblasts, pulmonary fibrosis involves other cell types, e.g., club cells (9) and macrophages (16), regulating the accumulation of fibroblasts and therefore the deposition of the extracellular matrix. As these cell types maintain autonomous circadian oscillations (2, 5), examination of circadian factors and mechanisms in the pulmonary fibrotic response is warranted.

The circadian clock operates as a cell-autonomous timing mechanism (17), allowing temporal segregation of both physiological and pathophysiological programs (18, 19). At the cellular level, the circadian clock consists of a transcription–translation feedback loop (20), in which the positive elements CLOCK and BMAL1 drive expression of 2 negative-feedback arms controlled by PERIOD/CRYPTOCHROME (PER/CRY) and the 2 paralogs, REVERBα and REVERBβ. In turn, these negative-feedback arms repress BMAL1/CLOCK heterodimer transactivation function (PER/CRY) or BMAL1 expression (REVERBα/β). The resulting 24-h oscillations in protein expression can be disrupted through environmental disruption (e.g., shift-work schedules) or genetic deletion of core clock components, producing inflammatory and metabolic phenotypes (5, 21, 22).

Here, we show that fibrotic mouse lungs exhibited amplified, but asynchronous, circadian rhythms with a dominant role for myofibroblasts. Disruption of the core clock protein REVERBα in fibroblastic cells resulted in exaggerated pulmonary fibrotic response to bleomycin in mice. In culture, REVERBα knockdown resulted in increased myofibroblast differentiation via the transcription factor TBPL1, through alteration of formation of integrinβ1 focal-adhesion expression. Furthermore, exposure to circadian stresses such as late chronotype, shift work, and altered sleep duration are all associated with IPF, and clock-gene expression is altered in IPF versus normal human lung. Targeting of REVERBα by a synthetic ligand repressed myofibroblast differentiation and collagen secretion in cultured fibroblasts and lung slices obtained from patients with lung fibrosis.

## Results

### Myofibroblasts Drive High-Amplitude, but Asynchronous, Circadian Oscillations in Fibrotic Lung.

**Fig. 1.**
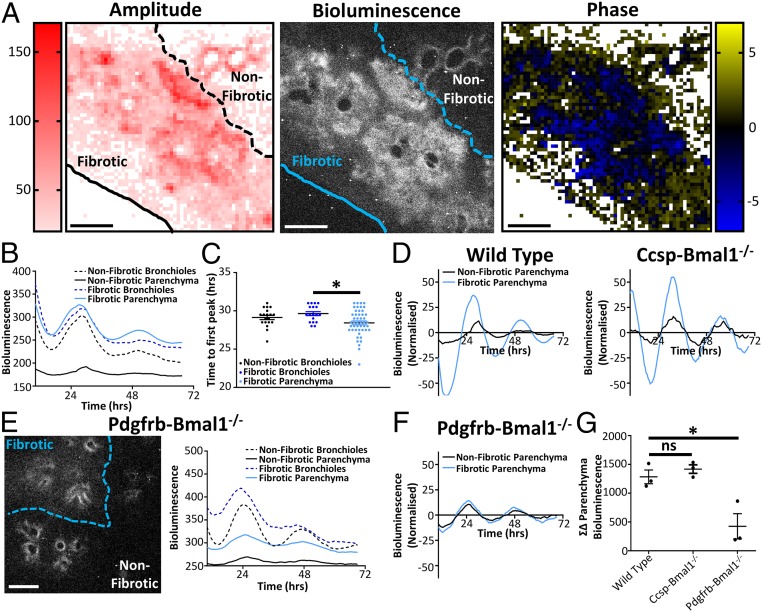
Asynchronous circadian oscillations occur in pulmonary fibrosis. (*A*) Bioluminescent image along with heat maps of amplitude and phase taken from the same PCLS obtained from a mPER2::luc mouse 14 d after in vivo bleomycin treatment (3 U/kg). Data are representative of 3 separate experiments. (Scale bars, 500 µm.) (*B*) Bioluminescent intensity plotted against time for both parenchyma and bronchioles in fibrotic and nonfibrotic regions (data are representative of 3 separate experiments). (*C*) Time to first peak for bronchioles and parenchyma in fibrotic and nonfibrotic areas. *P < 0.05 (ANOVA with post hoc Dunnett test using 18, 19, and 48 representative sections for healthy airways, fibrotic airways, and fibrotic parenchyma, respectively, in the lung slice). Data are representative of 3 separate experiments (mean ± SEM). (*D*) Bioluminescent intensity plotted against time (24-h moving average baseline subtracted) for the representative slices shown in *A* and Ccsp-Bmal1^−/−^ mice shown in *SI Appendix*, Fig. S2*A*. (*E*) Representative bioluminescent image along with bioluminescent intensity plotted against time for a PCLS 14 d after in vivo bleomycin treatment in the Pdgfrb-Bmal1^−/−^ mPER2::luc mouse (3 U/kg). Data are representative of 3 separate experiments. (Scale bar, 500 µm.) (*F*) Bioluminescent intensity plotted against time (24-h moving average baseline subtracted) for the Pdgfrb-Bmal1^−/−^ representative slice shown in *E*. (*G*) Difference in bioluminescence between fibrotic and nonfibrotic parenchyma over 3 d in PCLS from WT, Ccsp-Bmal1^−/−^, and Pdgfrb-Bmal1^−/−^ mice after in vivo bleomycin treatment (*n* = 3 animals). ns, not significant. *P < 0.05 (1-way ANOVA Dunnett post hoc test; mean ± SEM).

Precision-cut lung slices (PCLS) from transgenic mPER2::LUC mice (2) were used to track circadian oscillations in real time after bleomycin induction of fibrosis (Fig. 1 *A* and *B*, *SI Appendix*, Fig. S1 *A* and *B*, and Movie S1). Fibrotic areas were identified by loss of lung architecture in the bright-field image and confirmed with increased collagen deposition when the slices were fixed for histology (*SI Appendix*, Fig. S1 *A* and *B*). The amplitude of PER2 oscillations in the fibrotic areas was increased compared to nonfibrotic parenchyma lung (Fig. 1 *A*–*D*). This fibrotic parenchyma also had a greater degree of phase asynchrony compared to regions in the nonfibrotic parenchyma (Fig. 1*C*) but retained the same overall 24-h period (*SI Appendix*, Fig. S1*C*). One possible explanation may relate to changes in cell density in the fibrotic parenchyma. To explore this, PCLS were stained with Hoechst. There was a greater intensity of staining in fibrotic areas compared to nonfibrotic areas, but this did not correlate with bioluminescence (*SI Appendix*, Fig. S1 *D*–*F*). Another possible explanation is infiltration by a more rhythmic cell type; therefore, we deleted the essential core clock component BMAL1 (23) in both fibroblasts and club cells to ablate cell-autonomous rhythms. BMAL1 deletion in club cells (CCSP-Bmal1^−/−^), the main oscillatory cells in the lung (6), had no effect on the increased amplitude seen in fibrotic regions (Fig. 1 *D* and *G*, *SI Appendix*, Fig. S2*A*, and Movie S2). In contrast, BMAL1 deletion in pericyte/fibroblast lineage (Pdgfrb-Bmal1^−/−^) restored the amplitude of lung oscillations in fibrotic lung to levels measured in unaffected lung tissue (Fig. 1 *E*–*G*, *SI Appendix*, Fig. S2*B*, and Movie S3).

To test if fibrotic factors are capable of modifying circadian signals, lung slices and fibroblasts were treated with TGFβ. TGFβ induced changes in circadian phase (*SI Appendix*, Fig. S3*A*), with the magnitude of effect being dependent on both concentration and circadian phase (*SI Appendix*, Fig. S3 *B* and *C*). Lung physiology is also changed in fibrosis, resulting in an altered mechanoenvironment (24). Since tensile strength has recently been shown to play a key role in the regulation of tissue-based circadian rhythms (25), we investigated whether lung inflation, a cause of increased mechanical stretch, influenced circadian oscillations. Here, PER2 oscillation amplitude was increased in inflated lungs compared to noninflated controls, demonstrating that changes to the local mechanoenvironment may alter circadian oscillations (*SI Appendix*, Fig. S3*D*).

### REVERBα in Fibroblasts Suppresses the Development of Pulmonary Fibrosis.

**Fig. 2.**
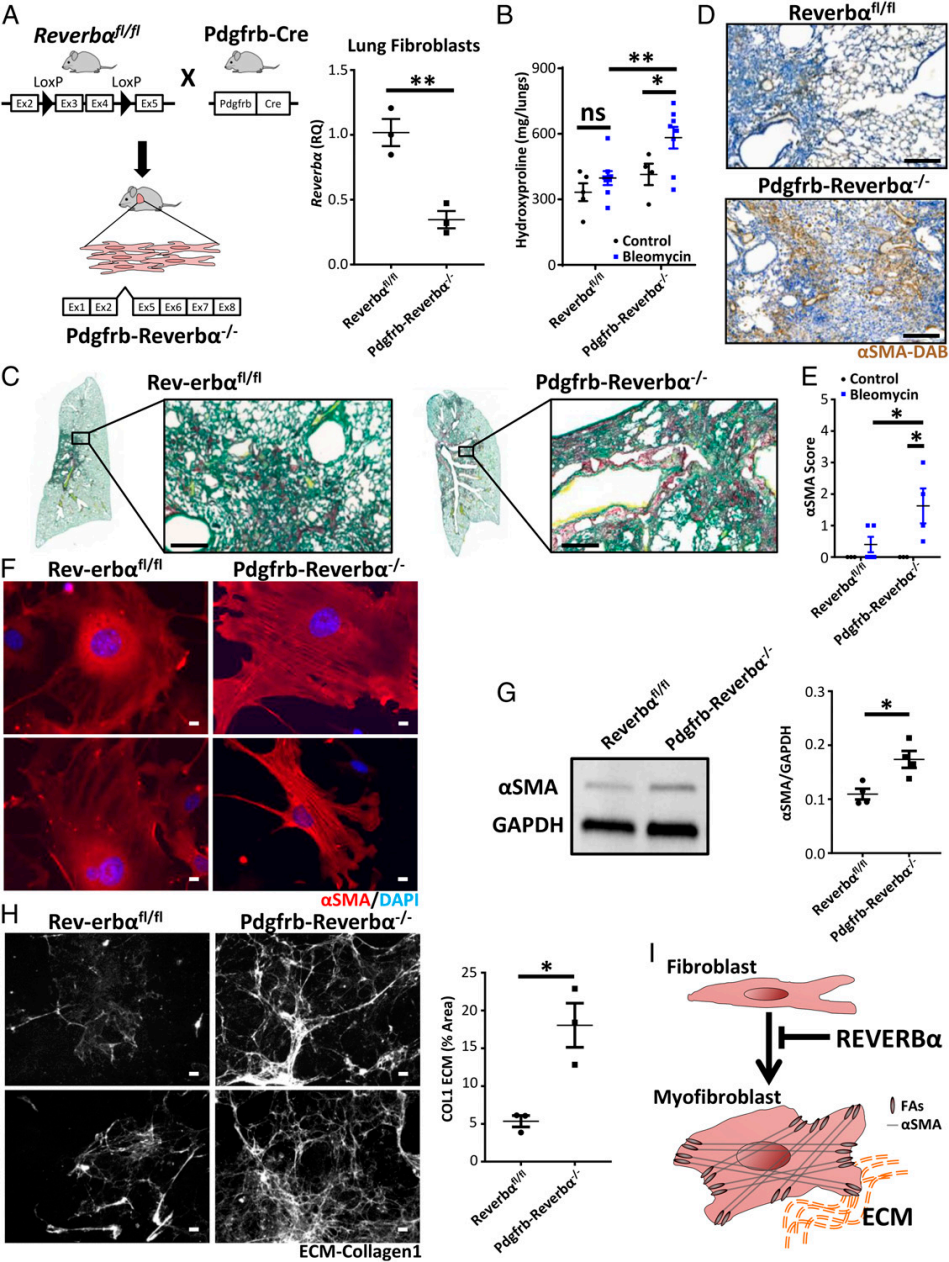
REVERBα alters susceptibility to pulmonary fibrosis through its effect on myofibroblast differentiation. (*A*) Schematic showing generation of Pdgfrb-Reverbα^−/−^ mice combined with qPCR analysis of *Reverbα* expression in lung fibroblasts (*n* = 3 animals). **P < 0.01 (Student *t* test; mean ± SEM). (*B*) Hydroxyproline measurement in lungs from Pdgfrb-Reverbα^−/−^ mice and littermate controls 28 d following challenge with intratracheal bleomycin (2 U/kg) or saline (*n* = 4 to 5 saline and 8 bleomycin per genotype). *P < 0.05; **P < 0.01 (2-way ANOVA Holm–Sidak post hoc test; mean ± SEM). (*C*) In a separate experiment, histology (Picrosirius red) of lungs was examined 28 d following challenge with intratracheal bleomycin (representative image from 4 animals treated with bleomycin per genotype). (Scale bars, 200 µm.) (*D*) Immunohistochemical staining of myofibroblasts (anti-αSMA, 3,3′-diaminobenzidine [DAB]) from Pdgfrb-Reverbα^−/−^ mice and littermate controls 28 d following intratracheal bleomycin challenge (representative image from 4 animals treated with bleomycin per genotype). (Scale bars, 200 µm.) (*E*) Histological scoring (grade 0 to 4) for the presence of αSMA staining 28 d following intratracheal bleomycin challenge (*n* = 3 saline and 4 to 5 bleomycin per genotype). *P < 0.05 (2-way ANOVA Holm–Sidak post hoc test; mean ± SEM). (*F* and *G*) Representative immunofluorescence images of primary lung fibroblast cultures from Pdgfrb-Reverbα^−/−^ mice and littermate controls showing intracellular αSMA (red) (*n* = 3 animals per genotype) (*F*) combined with a representative immunoblot and quantification of intracellular αSMA from primary lung fibroblast cultures (*n* = 4 animals per genotype) (*G*). *P < 0.05 (Student *t* test; mean ± SEM). DAPI, 4′,6-diamidino-2-phenylindole. (Scale bars in *F*, 10 µm.) (*H*) Representative collagen-1 ECM (extracellular matrix) images and quantification following culture of Pdgfrb-Reverbα^−/−^ and Reverbα^fl/fl^ primary lung fibroblasts (*n* = 3 animals per genotype). *P < 0.05 (Student *t* test; mean ± SEM). (Scale bars, 50 µm.) (*I*) Schematic illustrating the action of REVERBα in inhibiting fibroblast/myofibroblast differentiation. FAs, focal adhesions.

REVERBα is an orphan nuclear receptor and operates both as an essential core clock factor and as a major clock output pathway. Its function can be disrupted by deletion of its DNA-binding domain, and small molecular ligands are available to modulate activity. Therefore, we deleted the REVERBα DNA-binding domain (Fig. 2*A*), under Pdgfrb control (26). This resulted in an exaggerated fibrotic response (Fig. 2 *B* and *C*) and increased accumulation of αSMA-positive myofibroblasts in response to bleomycin (Fig. 2 *D* and *E*). Wild-type (WT) and transgenic mice did not differ in lung parameters following saline inoculation (Fig. 2 *B* and *E* and *SI Appendix*, Fig. S4 *A* and *B*). Importantly, REVERBα genetic disruption in myelomonocytic cells or bronchial epithelial cells did not affect the development of the fibrotic phenotype (*SI Appendix*, Fig. S4 *C* and *D*).

Characterization of primary fibroblasts explanted from Pdgfrb-Reverbα^−/−^ lungs ex vivo revealed increased expression of αSMA and increased secretion of collagen-1, markers of myofibroblast activation (Fig. 2 *F*–*H*). This indicates a fibroblast-intrinsic change driven by disruption of REVERBα, with culture on hard plastic providing the environmental trigger for initiation of the myofibroblast differentiation program (Fig. 2*I*).

### Knockdown of REVERBα in Vitro Enhances Myofibroblast Activation through the Transcription Factor TBPL1.

**Fig. 3.**
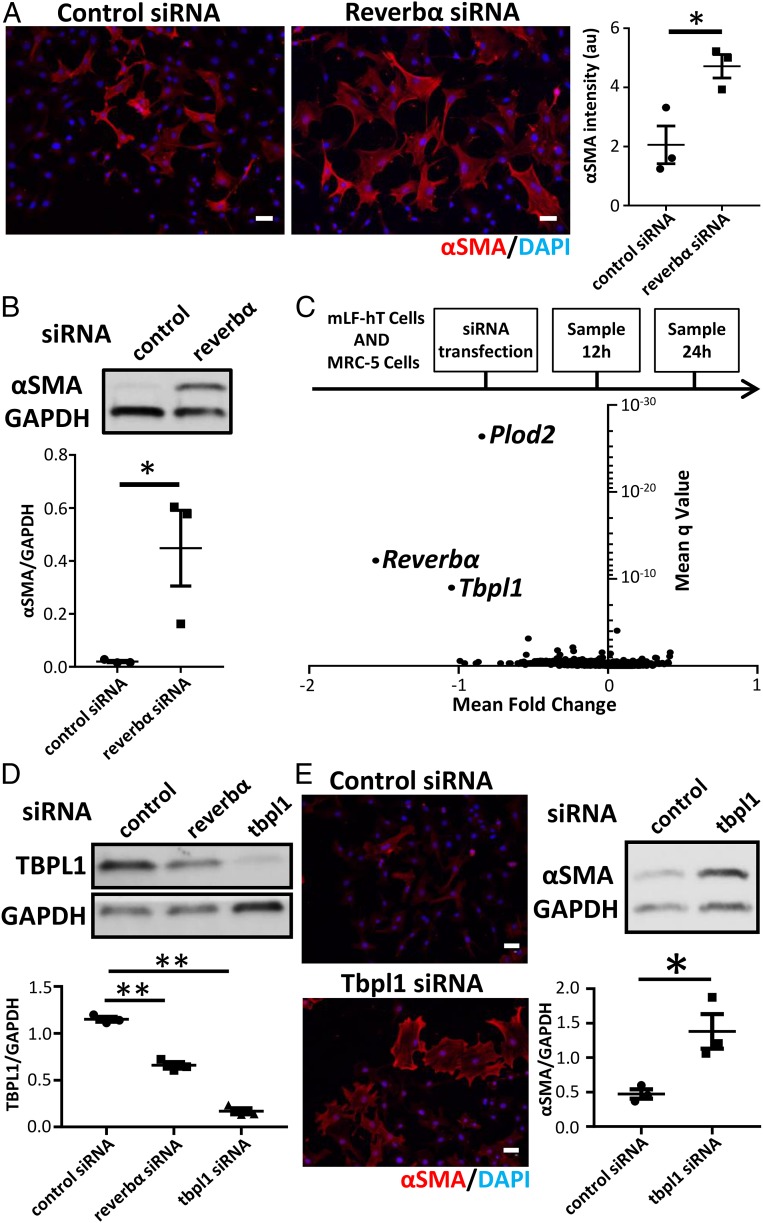
REVERBα alters myofibroblast differentiation via TBPL1. (*A*) Immunofluorescent staining and quantification for the myofibroblast marker αSMA after control (nontargeting) or *Reverbα* siRNA knockdown in mLF-hT cells (*n* = 3 separate transfections). *P < 0.05 (Student *t* test; mean ± SEM). (Scale bars, 50 µm.) (*B*) Immunoblot and densitometry for αSMA in MRC-5 cells after control (nontargeting) or *REVERBα* siRNA knockdown (representative immunoblot shown; *n* = 3 separate transfections). *P < 0.05 (Student *t* test; mean ± SEM). (*C*) Schematic of RNA-seq sample preparation. Control (nontargeting) or *Reverbα* siRNA knockdown was performed in 2 fibroblast cell lines (mLF-hT cells and MRC-5). Samples were collected for RNA-seq analysis 12 and 24 h after siRNA transfection from 3 separate transfections for each cell line per time point. Pooled analysis of all 4 different RNA-seq experimental conditions is shown by a volcano plot (mean fold change plotted against mean q-value). (*D*) Immunoblot of TBPL1 following control (nontargeting) or *Reverbα* or *Tbpl1* siRNA knockdown in mLF-hT cells (a representative immunoblot is shown; *n* = 3 separate transfections). **P < 0.01 (Student *t* test; mean ± SEM). (*E*) Representative immunofluorescence and immunoblotting for αSMA after control (nontargeting) or *Tbpl1* siRNA knockdown in mLF-hT cells (a representative immunoblot is shown; *n* = 3 separate transfections). *P < 0.05 (Student *t* test; mean ± SEM). (Scale bars, 50 µm.) DAPI, 4′,6-diamidino-2-phenylindole.

Next, we set out to identify REVERBα gene targets using small interfering RNA (siRNA) knockdown of REVERBα in both mouse and human lung fibroblast cell lines (*SI Appendix*, Fig. S5*A*). REVERBα knockdown resulted in myofibroblast activation in lung fibroblast cells (Fig. 3 *A* and *B* and *SI Appendix*, Fig. S5 *B*–*D*). Although many genes were regulated by REVERBα knockdown, only 3 (including *Reverbα*) were consistently repressed at both time points (12 and 24 h) and in both cell lines (Fig. 3*C* and *SI Appendix*, Fig. S5 *E* and *F*). One was *Plod2*, a proline hydroxylase required for collagen processing. The second was *Tbpl1*, a relatively uncharacterized transcription factor. Knockdown of either PLOD2 or TBPL1 did not affect *REVERBα* expression (*SI Appendix*, Fig. S6*A*). In addition, knockdown of PLOD2 repressed αSMA expression, therefore making it an unlikely downstream mediator of the REVERBα effect (*SI Appendix*, Fig. S6*B*). Therefore, we turned to TBPL1 and verified loss of protein expression with REVERBα knockdown (Fig. 3*D*). Knockdown of TBPL1 caused a similar induction of αSMA expression to that seen with REVERBα knockdown (Fig. 3*E*), suggesting that REVERBα and TBPL1 may lie on the same pathway.

### REVERBα and TBPL1 Regulate Integrinβ1 Expression.

**Fig. 4.**
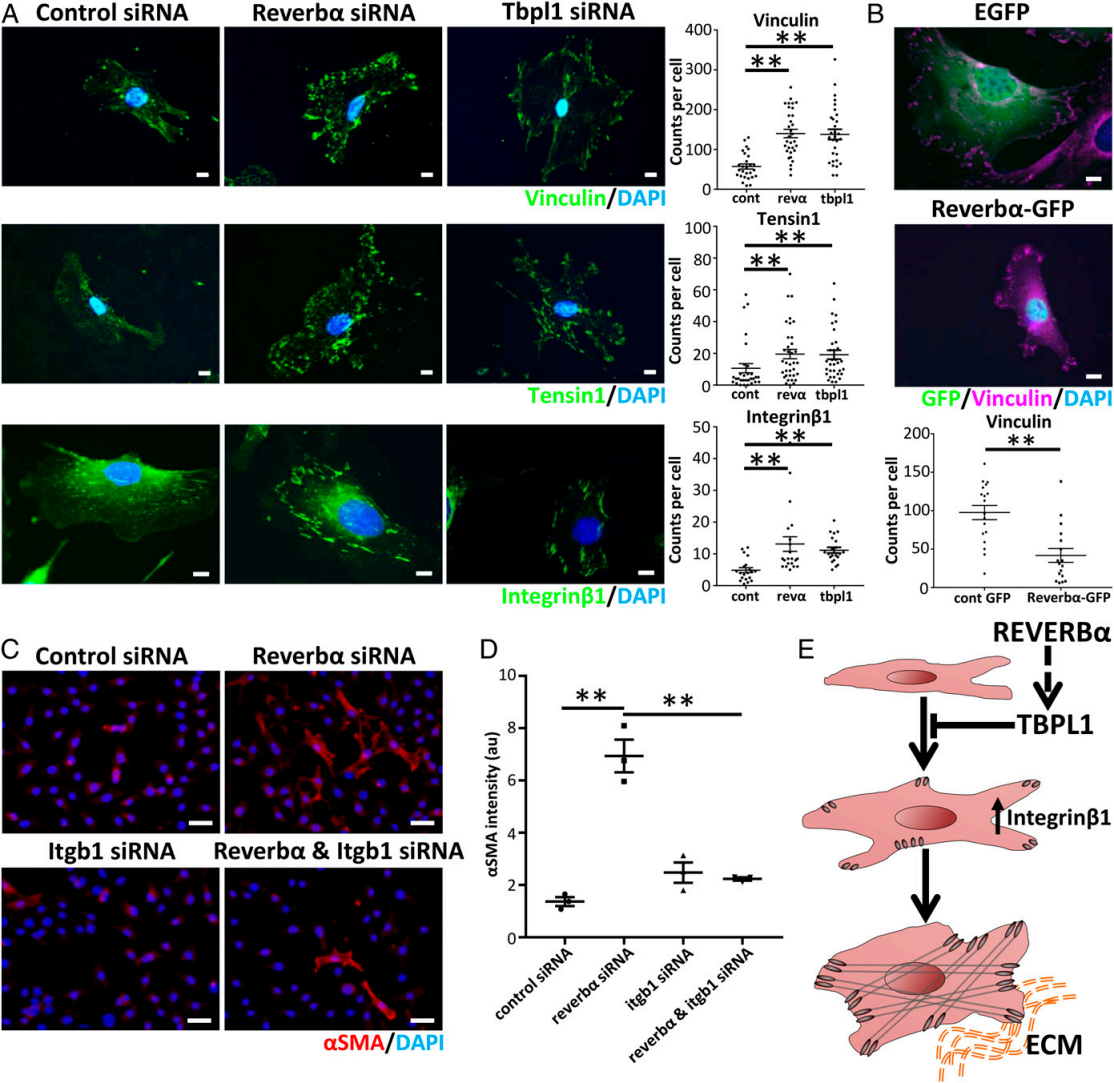
REVERBα and TBPL1 affect myofibroblast differentiation through changes in integrinβ1 expression. (*A*) Representative immunofluorescent images and quantification per cell of vinculin, tensin1, and integrinβ1 following siRNA knockdown of *Reverbα* or *Tbpl1* compared to control (nontargeting) siRNA in mLF-hT cells (*n* = 3 separate transfections). **P < 0.01 (1-way ANOVA post hoc Dunnett test; mean ± SEM). Dots represent individual cells from 3 transfections. cont, control siRNA; revα, *Reverbα* siRNA. (Scale bars, 10 µm.) (*B*) Representative immunofluorescence image after mLF-hT cells have been transfected with REVERBα-GFP plasmid or an empty-GFP plasmid. Cells were stained for GFP, vinculin, and nuclei (4′,6-diamidino-2-phenylindole [DAPI]) (*n* = 3 separate transfections). **P < 0.01 (Student *t* test; mean ± SEM). Dots represent individual cells from 3 transfections with the focal-adhesion number being quantified per cell. (Scale bars, 10 µm.) (*C*) Representative immunofluorescence images. (Scale bars, 50 µm.) (*D*) Quantification of the myofibroblast marker αSMA following dual siRNA knockdown (control or *Reverbα* in the presence or absence of *Itgb1*) in mLF-hT cells (*n* = 3 separate transfections). **P ≤ 0.01 (1-way ANOVA post hoc Dunnett test; mean ± SEM). (*E*) Schematic demonstrating how both REVERBα and TBPL1 regulate Integrinβ1, which in turn affects myofibroblast differentiation. ECM, extracellular matrix.

To decipher how REVERBα and/or TBPL1 suppress myofibroblast activation in fibrotic lungs and in the stiff cell culture environment, we focused on focal adhesions, crucial mechanotransduction elements that control myofibroblast activation (27). Knockdown of either REVERBα or TBPL1 resulted in increases of both size and number of vinculin/tensin1-positive focal-adhesion complexes (Fig. 4*A* and *SI Appendix*, Fig. S7 *A*–*C*). This increase in size suggests progression to the supermature focal adhesions involved in myofibroblast differentiation (27). In contrast, overexpression of REVERBα or TBPL1 caused the opposite effect (Fig. 4*B* and *SI Appendix*, Fig. S7 *D* and *E*). Integrinβ1, the common subunit of all collagen1-binding integrins, has previously been linked to myofibroblast activation in the liver (28), lung (29), and scleroderma (30). Knockdown of either REVERBα or TBPL1 resulted in an increase in both size and number of integrinβ1-positive focal-adhesion complexes (Fig. 4*A* and *SI Appendix*, Fig. S7*A*). Furthermore, knockdown of integrinβ1 prevented the induction of αSMA seen in fibroblasts cultures subjected to REVERBα knockdown (Fig. 4 *C* and *D*), highlighting the requirement for integrinβ1 for REVERBα-mediated myofibroblast activation (Fig. 4*E*).

### Circadian Factors Are Associated with Pulmonary Fibrosis in Humans.

**Fig. 5.**
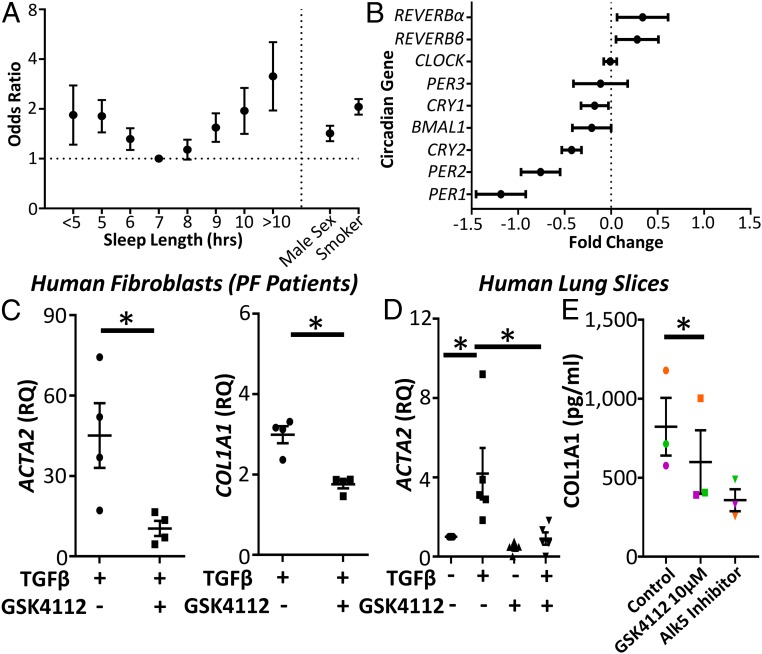
Circadian factors are associated with IPF, where a REVERB ligand represses collagen secretion. (*A*) Odds ratio (OR) for the association between pulmonary fibrosis and sleep duration (OR ± 95% confidence interval (CI); logistic regression; *n* = 500,074 subjects from the UK Biobank). (*B*) Changes in clock-gene expression in IPF compared to control subjects from a previously published genome array (GSE47460) (fold change ± 95% confidence interval; *n* = 90 controls and 98 patients with IPF). (*C*) qPCR for αSMA (*ACTA2*) and Collagen1 (*COL1A1*) following TGFβ stimulation (2 ng/mL) in primary human lung fibroblasts obtained from patients with pulmonary fibrosis in the presence or absence of GSK4112 (10 μM) (*n* = 4 fibrotic patients). *P < 0.05 (Student *t* test; mean ± SEM). (*D*) qPCR for αSMA (*ACTA2*) expression following treatment with TGFβ (2 ng/mL) and GSK4112 (10 μM) in human PCLS (*n* = 5 patients). *P < 0.05 (Student *t* test; mean ± SEM). (*E*) Enzyme-linked immunosorbent assay analysis of secreted collagen-1 in TGFβ-stimulated PCLS obtained from 3 patients with IPF treated with the REVERB ligand GSK4112 (10 μM) and an Alk5 inhibitor (1 μM) as positive control (*n* = 3). *P < 0.05 (paired Student *t* test; mean ± SEM).

Several human factors have been associated with circadian or sleep-deprivation strain, including evening chronotype, shift work, and sleep duration. We therefore investigated whether these factors were associated with pulmonary fibrosis in the UK Biobank (*n* = 500,074) (31). Following adjustment for known risk factors for pulmonary fibrosis (body mass index, smoking, sex, and age), short or long sleep duration (<7 h or >7 h) were associated with pulmonary fibrosis (Fig. 5*A* and *SI Appendix*, Tables S1 and S2), with the size of the odds ratio (OR) being greater than the established risk factors of age, sex, or smoking in the multivariable model. Shift work (OR: 1.353; 95% confidence interval [CI]: 1.069 to 1.710) and evening chronotype (OR: 1.040; 95% CI: 1.001 to 1.080) were also associated with pulmonary fibrosis (*SI Appendix*, Tables S3–S6) by a smaller degree; however, this is comparable to other diseases where these variables are risk factors (32–34).

### Disordered Clock-Gene Expression Occurs in Idiopathic Pulmonary Fibrosis.

To look for evidence of circadian-clock disruption in IPF, we analyzed lung gene expression in a previously published microarray from the lung genomics research consortium (35). Comparison with normal lung revealed significant differences in *PER1*/*2*, *CRY 2*, and *REVERBα*/*β* (Fig. 5*B*), all encoding components of the negative-feedback arm of the core circadian clock. In addition, *TBPL1* was up-regulated in pulmonary fibrosis, correlating with *REVERBα* expression (*SI Appendix*, Fig. S8*A*).

### A REVERB Ligand Inhibits Myofibroblast Differentiation and Represses Collagen Secretion in Tissue from Pulmonary Fibrotic Patients.

Finally, we tested whether a REVERBα ligand could repress pulmonary fibrosis. The well-characterized REVERBα agonist GSK4112 (36) repressed TGFβ-induced expression of αSMA (*ACTA2*) and collagen-1 (*COL1A1*) in primary human lung fibroblasts from patients with pulmonary fibrosis (Fig. 5*C*). Similarly, TGFβ induction of αSMA and collagen 1 transcription was prevented by GSK4112 treatment in precision-cut human lung, organotypic slice cultures from healthy control subjects (Fig. 5*D*). Finally, we studied the effects of this ligand in PCLS from IPF patients along with an Alk5 inhibitor, known to inhibit Col1a1 secretion (37). GSK4112 repressed Col1a1 secretion (Fig. 5*E*) in a similar manner to the Alk5 inhibitor.

## Discussion

Pulmonary fibrosis is an intractable and fatal disease. We have previously identified the lung as a highly circadian organ and that responses to environmental insults are regulated and shaped by the circadian clock. Therefore, we analyzed mouse lung fibrosis, finding newly-emergent, and strong circadian oscillations driven by fibroblasts. The prevalent profibrotic growth factor TGFβ was capable of transmitting timing information to recipient cells, and disruption to the core circadian clock in fibroblasts increased fibrotic response to bleomycin instillation. In vitro analysis identified a circuit linking the core clock through REVERBα, to TBPL1, and the focal adhesions important for myofibroblast activation. In human IPF lung tissue, pharmacological targeting of the clock impacted a surrogate measure of fibrotic progression, and we found an association between sleep duration, which is a product of the circadian clock, and risk of pulmonary fibrosis.

Several studies have found that circadian responses in the lung are gated through club cells (2) or macrophages (5). A previous report suggested that the acute inflammatory phase (7 d) of the bleomycin response lay under circadian control (4); therefore, we investigated circadian function in developing fibrosis. Surprisingly, there were higher-amplitude circadian oscillations in fibrotic tissue compared to normal lung tissue, but these oscillations were asynchronous, suggesting a possible role for circadian mechanisms (38). The process of fibrosis involves several different cell types including club cells, macrophages, and fibroblasts (9), but as genetic deletion of the only nonredundant circadian gene *Bmal1* to the pericyte lineage stopped the emergent oscillations, the importance of fibroblasts was established. The importance of the fibroblast was further confirmed by finding that REVERBα deletion in these cells impacted the fibrotic response, but disruption in other cell types was without effect. These results build on previous discoveries showing that circadian oscillations in fibroblasts are robust (39) and alter wound-healing (40, 41).

Mechanistically, knockout or knockdown of REVERBα promoted myofibroblast activation in vitro, with the reverse effects seen with REVERBα overexpression. Analysis of REVERBα gene targets revealed striking enrichment for a single transcription factor, TBPL1, and the emergence of a coherent pathway converging on increased formation of integrinβ1 focal-adhesion complexes. To the best of our knowledge, TBPL1 has not been previously implicated in fibrotic disease, but we found its expression elevated in human IPF tissue. This and the elevated REVERBα expression are an apparent paradox, as both proteins inhibit myofibroblast activation. Therefore, we hypothesize that the increase in both TBPL1 and REVERBα in fibrotic tissue results from tissue compensation in response to fibrosis, making it a promising therapeutic pathway. Integrinβ1 emerged as the final effector, and the focal adhesions associated with it have previously been established (28) to be important for myofibroblast activation.

We have successfully used large-scale human cohorts, such as the UK Biobank, to explore connections between measures of circadian strain (shift work, chronotype, and sleep) and prevalent disease (34, 42, 43). Low-prevalence diseases such as pulmonary fibrosis present unique challenges. To address this, we identified people with pulmonary fibrosis participating in the UK Biobank (31) and linked them with information from Hospital Episode Statistic data (44). Importantly, patients were not screened for pulmonary fibrosis on enrolling in the Biobank; therefore, we cannot comment on causality, but it is clear that short sleep length is associated with pulmonary fibrosis, and this is as least as strong as existing risk factors for this disease (45), indicating potential clinical relevance. An association with long sleep duration was also found that may be biological (46) or due to confounders (47).

We, and others, have developed tool compounds capable of activating REVERBα (48, 49). These permit extension of our studies to primary human tissue, which is hard to genetically manipulate. Here, we show a marked inhibition of the myofibroblast phenotype, blunted fibrotic response to TGFβ stimulation and reduced collagen-1 secretion in IPF PCLS. We, and others, have also shown that these compounds have off-target effects (48, 50), and therefore it is reassuring that knockdown and overexpression of REVERBα in human fibroblasts had similar effects on both our mice studies and also the ligand. The recent publication (50) that the only ligand with suitable pharmacokinetics for in vivo experiments has significant off-target effects combined with the lack of translation from the mouse bleomycin model to the clinic (51) precludes an in vivo mouse experiment to confirm its therapeutic effectiveness.

Taken together, our results identify a surprising and potent role for the core circadian-clock factor REVERBα in the activation of myofibroblasts via a pathway incorporating a poorly characterized transcription factor, TBPL1, which affects the development of pulmonary fibrosis.

## Methods

### Mouse Lines.

mPER2::luc transgenic mice were previously described (52). The Rev-erbα^fl/fl^ mouse (Rev-erbαDBD^m^) and Cre drivers targeting club cells (CCSP^icre^) and myeloid cells (Lysm^cre^) are as previously described (1). The PDGFRβ^cre^ mouse was a kind gift from N.C.H. and has been previously described (14). The Bmal1^fl/fl^ mouse has been previously described (2).

### Cell Culture.

MRC-5 cells or mLF-hT cells (16) were cultured in Dulbecco modified Eagle media.

### In Vivo Bleomycin.

Male mice were challenged intratracheally with bleomycin (Sigma) or saline (vehicle).

### Bioluminescence Microscopy.

Organotypic PCLS were prepared as described before (2). Bioluminescence images were obtained using a 2.5× objective (Zeiss) and captured using a cooled Andor iXon Ultra camera over a 30-min integration period.

### Immunofluorescence.

For αSMA staining, cells in 35-mm dishes were fixed in 4% paraformaldehyde (PFA)/0.2% Triton X-100, followed by ice cold methanol fixation. For focal-adhesion proteins, cells were exposed to ice-cold cytoskeleton buffer (53) for 10 min followed by 4% PFA fixation for a further 10 min.

### RNA Sequence.

siRNA-transfected mLF-hT and Mrc5 cells were lysed, and RNA was extracted using the ReliaPrep RNA miniprep system. RNA was sequenced on an Illumina HiSEq 4000. Analysis of these data was performed using the Ingenuity Pathway Analysis software (Qiagen).

### UK Biobank.

The UK Biobank was accessed January 2019, and the data were combined with the Hospital Episode data set (54). Subjects were excluded a priori if they took sleep-altering medication or had obstructive sleep apnea.

### Microarray Analysis.

Geo2R (55) was used to analyze GSE47460 generated by the Lung Genomics Research Consortium (56).

### Human PCLS.

PCLS were cut at 400 µm on a vibrating microtome. TGFβ, GSK4112, or vehicle (dimethyl sulfoxide) treatments were performed each day with the slices being lysed after 4 d for qPCR analysis or 7 d for supernatant analysis. Additional methods can be found in *SI Appendix*.

### Data Availability.

The RNA-sequence (RNA-seq) data have been deposited in the ArrayExpress Archive of Functional Genomics Data (accession no. E-MTAB-8499) (57). Matlab code for circadian analysis of Fig. 1 has been deposited in Mendeley database (doi:10.17632/5wr5s3w4s7.1) (58).

## Supplementary Material

Supplementary File

Supplementary File

Supplementary File

Supplementary File
